# S-equol Modulates T3-Induced Transcription and Neurite Outgrowth in Neuronal Cells

**DOI:** 10.3390/ijms27073253

**Published:** 2026-04-03

**Authors:** Yuki Fujiwara, Winda Ariyani, Ayane Ninomiya, Wataru Miyazaki, Ririka Ota, Izuki Amano, Noriyuki Koibuchi

**Affiliations:** 1 Department of Integrative Physiology, Gunma University Graduate School of Medicine, Maebashi 371-8511, Japan; y-fujiwara@gunma-u.ac.jp (Y.F.); winda@gunma-u.ac.jp (W.A.); akninomiya.sci@gmail.com (A.N.); miya@hirosaki-u.ac.jp (W.M.); 2 Department of Bioscience and Laboratory Medicine, Hirosaki University Graduate School of Health Science, Hirosaki 036-8564, Japan; 3 School of Medicine, Gunma University, Maebashi 371-8511, Japan; ririka.o1231@gmail.com; 4 Ota College of Medical Technology, Ota 373-0812, Japan

**Keywords:** thyroid hormone, S-equol, neuronal morphogenesis

## Abstract

Thyroid hormones (THs) and estrogen (E2) play essential roles in neuronal differentiation and plasticity during brain development. S-equol, a plant-derived isoflavone metabolite, is a selective E2 receptor (ER) ligand that exhibits neurotrophic effects; however, its interaction with TH receptor (TR) signaling remains unclear. In this study, we investigated the effects of S-equol on TR*β*-associated transcriptional activity and neuronal morphogenesis in mouse neuroblastoma-derived Neuro-2a cells or rat C6 glioma cells. Luciferase reporter assays demonstrated that S-equol significantly enhanced T3-induced TR*β* transcriptional activity in a concentration- and time-dependent manner. Additionally, exposure to S-equol or T3 alone promoted neurite outgrowth and wound closure, whereas co-exposure to both compounds resulted in a more significant enhancement of these processes. Furthermore, mRNA expression levels of synapse-related genes (*Dlg4*, *Syn1*, *Syp*, *Camk2b*, and *Bdnf*) were significantly increased by S-equol co-exposure in the presence of T3. In silico docking analysis revealed that S-equol exhibited moderate to high binding affinity for TR*β* (−8.7 kcal/mol), ER*α*, and ER*β*, suggesting a structural basis for TR–ER crosstalk. Collectively, these findings indicate that S-equol functions as a dual-acting modulator that may modulate T3 signaling involving TR–ER interaction. Although S-equol may exert beneficial effects on neurodevelopment, it may also act as an endogenous endocrine modulator that alters the fine regulation of TH action during development, warranting careful evaluation from physiological and toxicological perspectives.

## 1. Introduction

Thyroid hormones (THs) are essential endocrine factors that regulate diverse physiological processes, including neural development, axonal elongation, and metabolic control, from the fetal stage through adulthood [1]. These actions are primarily mediated by nuclear TH receptors (TRs), whereby TR*α* and TR*β* bind to TH response elements (TREs) on target genes and regulate transcription in a T3-dependent manner [2].

Estrogen (E2) is also a key hormone involved in neuronal differentiation, synapse formation, and the maintenance of neural plasticity, exerting its effects through E2 receptors (ERs) to regulate the expression of numerous neural-related genes [3,4]. TRs and ERs belong to the nuclear receptor superfamily and share structurally similar DNA-binding domains and common coactivator complexes, allowing potential interference and interaction between their signaling pathways [5,6].

Indeed, studies using the neuroblastoma-derived SK-N-BE(2)-C cell model have demonstrated that T3 enhances E2 response element (ERE)-dependent transcription [7]. Because this effect is attenuated by mitogen-activated protein kinase (MAPK) pathway inhibition, a functional linkage between genomic and non-genomic signaling pathways has been suggested. In pituitary tumor-derived GH3 cells, T3 treatment increased ER expression and augmented ERE-dependent transcriptional activity [8]. These findings indicate that T3 can modulate transcriptional regulation via the ER pathway and support the existence of TR–ER crosstalk. Such hormonal interactions exert either stimulatory or inhibitory effects, depending on cell type and promoter context [9,10].

In addition to transcriptional regulation mediated by nuclear receptors, TH and E2 are known to elicit non-genomic actions through membrane-associated receptors and intracellular signaling pathways. TH induces rapid signaling via integrin αvβ3 [11], whereas E2 regulates intracellular calcium dynamics and MAPK activity through the membrane-associated receptor, G protein-coupled E2 receptor 1 (GPER1) [12]. This complex network of receptor-mediated signaling is particularly susceptible to modulation by exogenous chemicals and has been implicated as a contributing factor in endocrine disruption [13].

Plant-derived isoflavones constitute a group of compounds with E2-like activity, among which S-equol, a gut microbial metabolite of daidzein, exhibits high affinity for ERs [14,15]. S-equol promotes neurite outgrowth and neuronal differentiation and exerts neuroprotective effects [16,17]. Furthermore, our study group has previously demonstrated that isoflavones, including genistein, daidzein, and S-equol, activate TR-dependent transcription [18], and that S-equol induces neurite formation through nuclear and membrane-associated receptors [16,17,19]. These findings suggest that S-equol may act not only on ER signaling but also on the TR pathway. However, studies focusing on the modulation of TR-dependent transcription by E2-like compounds remain limited. In particular, the effects of S-equol on TR-dependent transcriptional activity and neuronal morphogenesis in neuroendocrine and glial model systems, such as Neuro-2a and C6 cells, have not been fully elucidated. The aim of this study was to investigate the effects of S-equol on T3-responsive transcriptional activity and neuronal morphogenesis, and to explore the potential interaction between thyroid hormone receptor (TR) and estrogen receptor (ER) signaling pathways.

## 2. Results

### 2.1. Concentration- and Time-Dependent Enhancement of TR*β*-Associated T3-Induced Transcriptional Activity by S-equol

**Figure 1:**
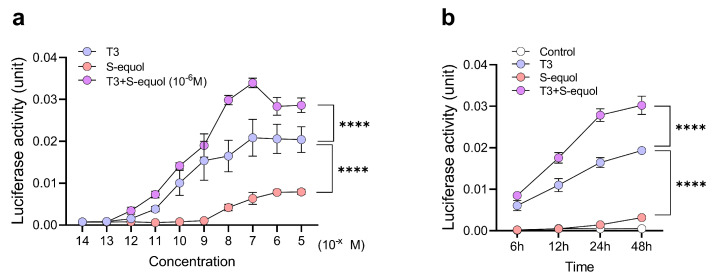
Concentration- and time-dependent effects of S-equol on TR*β*-associated T3-induced transcriptional activity in Neuro-2a cells. (**a**) Neuro-2a cells transfected with a human TR*β*–TRE–luciferase reporter were exposed for 24 h to T3 (10^−14^ to 10^−4^ M), S-equol (10^−14^ to 10^−4^ M), or co-exposure to S-equol (10^−6^ M) with varying concentrations of T3. Luciferase activity was normalized to control and expressed as relative activity. (**b**) Time-course analysis of luciferase activity following exposure to T3 (10^−7^ M), S-equol (10^−6^ M), or their combination. Data are presented as mean ± standard error of the mean (SEM). Statistical significance was assessed using two-way analysis of variance (ANOVA) followed by the Bonferroni test. **** *p* < 0.0001 vs. the T3-treated group.

Neuro-2a cells were transfected with a human TR*β*–TRE–luciferase reporter plasmid and exposed for 24 h to T3 (10^−14^–10^−5^ M), S-equol (10^−14^–10^−5^ M), or co-exposure to T3 (10^−14^–10^−5^ M) in the presence of S-equol (10^−6^ M) (Figure 1a). T3 alone induced a concentration-dependent increase in transcriptional activity. Although the effect was weaker than that observed with T3, S-equol alone also increased transcriptional activity in the concentration range of 10^−8^–10^−5^ M. Similarly, co-exposure to T3 and S-equol significantly enhanced luciferase activity compared with T3 alone within the same concentration range (10^−8^–10^−5^ M) (by Bonferroni test).

Time-dependent effects of co-exposure to T3 (10^−7^ M) and S-equol (10^−6^ M) were further examined (Figure 1b). The T3 + S-equol group consistently exhibited higher luciferase activity than the T3- or S-equol-alone groups, with particularly pronounced differences observed at 24–48 h. Although S-equol alone induced a modest increase in activity compared with the control group, this effect was markedly smaller than that induced by T3 or by co-exposure to T3 and S-equol. These results indicate that S-equol enhances T3-induced transcriptional activity in a time-dependent manner and that co-exposure accelerates the onset and attainment of maximal transcriptional responses.

### 2.2. Effects of S-equol and T3 on Neuronal Morphology

**Figure 2:**
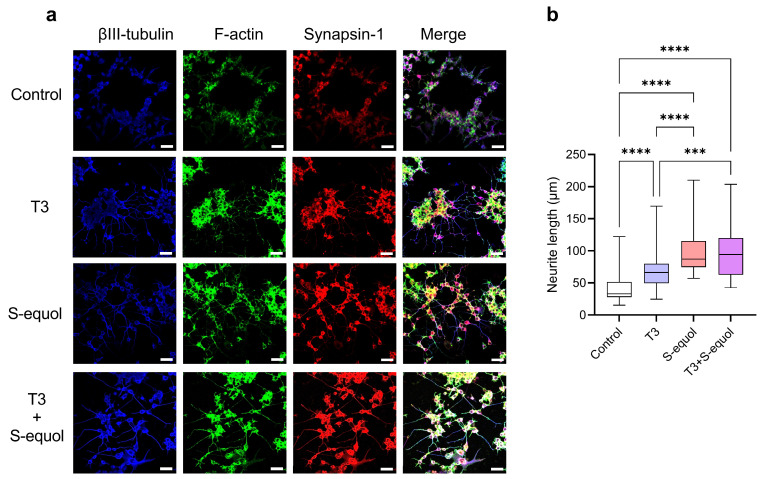
Effects of S-equol and T3 on neurite outgrowth in Neuro-2a cells. (**a**) Representative fluorescence images and quantitative analysis of neurite outgrowth following exposure to T3 (10^−8^ M), S-equol (10^−8^ M), or their combination (T3 10^−9^ M + S-equol 10^−8^ M). βIII-tubulin (blue) was used as a neuronal marker, F-actin (green) was used to visualize the cytoskeleton, and Synapsin I (red) was used as a presynaptic marker. Scale bar = 100 μm. (**b**) Quantification of neurite length based on βIII-tubulin-positive neurites. Data are shown as mean ± SEM. Statistical analysis was performed using one-way ANOVA followed by Bonferroni test. *** *p* < 0.001, **** *p* < 0.0001.

To determine whether changes in TR*β*-associated transcriptional activity induced by S-equol affect neuronal differentiation-related functions, neurite outgrowth was evaluated in Neuro-2a cells (Figure 2). Neuro-2a cells were exposed to T3 (10^−8^ M), S-equol (10^−8^ M), or co-exposure to both compounds (T3 10^−9^ M + S-equol 10^−8^ M), and neurite outgrowth was assessed (Figure 2a). Quantification of neurite length based on βIII-tubulin immunostaining revealed significant neurite elongation in the T3-treated and T3 + S-equol co-exposure groups compared with the control group (Figure 2b). Moreover, neurite length in the T3 + S-equol co-exposure group was significantly greater than that in the T3-alone group, suggesting that S-equol enhances T3-associated neurite formation.

### 2.3. Effects of S-equol and T3 on Wound Closure

**Figure 3:**
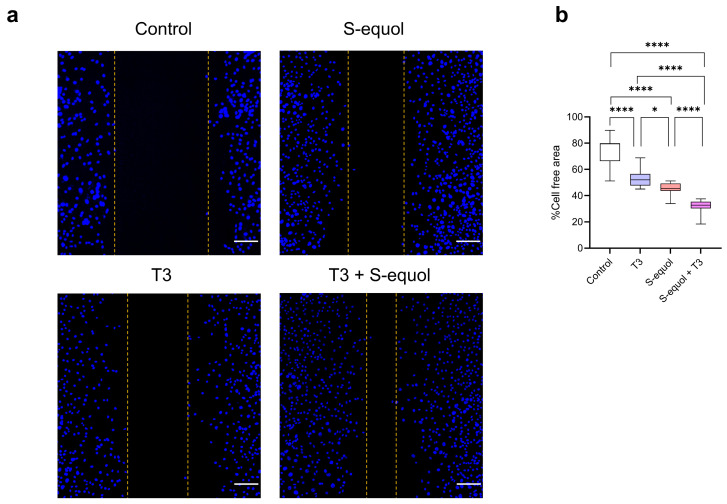
Effects of S-equol and T3 on neuronal morphology and wound closure in C6 cells. (**a**) Representative images of Neuro-2a cells following exposure to T3 (10^−8^ M), S-equol (10^−8^ M), or their combination (T3 10^−9^ M + S-equol 10^−8^ M) after wound healing (scratch) assay. Cell nuclei were visualized by Hoechst staining (blue). Scale bar = 100 µm. (**b**) Quantitative analysis of the effects of S-equol and T3 on wound closure measured by wound healing assay. Data are shown as mean ± SEM. Statistical analysis was performed using one-way ANOVA followed by Bonferroni test. * *p* < 0.05, **** *p* < 0.0001.

A wound healing (scratch) assay performed using C6 clonal cells under the same condition as Figure 2 demonstrated significant differences among groups by one-way analysis of variance (Figure 3). Bonferroni multiple comparison test revealed a significant increase in wound closure, reflecting cell motility-related processes from the control group to the T3-treated group, the S-equol-alone group, and the T3 + S-equol co-exposure group, with the strongest effect observed under co-exposure conditions. Immunocytochemical analysis further showed detectable staining of Rho GTPase family members, including Cdc42, Rac1/2/3, and RhoA, in cells treated with S-equol, T3, or their combination, whereas little to no signal was observed in control cells (Supplementary Figures S1–S3).

### 2.4. Effects of S-equol and T3 on the Expression of Synapse-Related Genes

**Figure 4:**
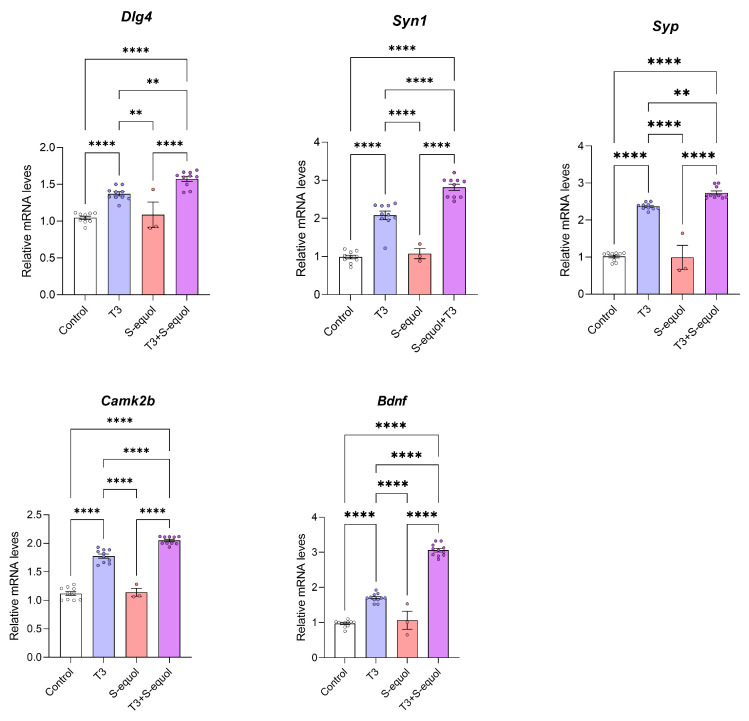
Effects of S-equol and T3 on the expression of synapse-related genes in Neuro-2a cells. Neuro-2a cells were treated for 24 h with T3 (10^−8^ M), S-equol (10^−8^ M), or their combination (T3 10^−9^ M + S-equol 10^−8^ M), and mRNA expression levels of *Dlg4*, *Syn1*, *Syp*, *Camk2b*, and *Bdnf* were quantified using quantitative polymerase chain reaction. Data are shown as mean ± standard error of the mean. Statistical significance was assessed using one-way analysis of variance followed by the Bonferroni test. ** *p* < 0.01, **** *p* < 0.0001.

To examine the effects of S-equol and T3 on the expression of synapse formation-related genes, Neuro-2a cells were treated for 24 h with T3 (10^−8^ M), S-equol (10^−8^ M), or co-exposure to both compounds (T3 10^−9^ M + S-equol 10^−8^ M), and mRNA expression levels of *Dlg4*, *Syn1*, *Syp*, *Camk2b*, and *Bdnf* were quantified using quantitative polymerase chain reaction (Figure 4). These genes are representative markers involved in synapse formation and neuronal plasticity. T3 treatment alone significantly increased the expression of all analyzed genes compared with the control group. In contrast, S-equol alone did not significantly affect the expression levels of any of these genes. Notably, co-exposure to T3 and S-equol resulted in a further significant increase in gene expression compared with T3 treatment alone, with consistent enhancement observed across all targets.

### 2.5. Comparative Analysis of the Binding Affinity of S-equol to TH and ERs

To clarify the binding properties of S-equol to TH and ERs, docking analyses were performed using the ligand-binding domain structures of each receptor. First, binding free energies of the reference ligands T3 and E2 were calculated (Table 1). T3 exhibited mean binding energies of −9.4 and −9.6 kcal/mol for TR*α* and TR*β*, respectively, confirming its high affinity for both receptors. In contrast, E2 showed binding energies of −8.6 and −8.5 kcal/mol for ER*α* and ER*β*, respectively, indicating selective high-affinity binding to ERs.

Subsequent calculation of binding energies for S-equol revealed values of −8.7 kcal/mol for TR*β*, −8.4 kcal/mol for ER*α*, and −8.2 kcal/mol for ER*β*, indicating moderate to high affinity for all receptors examined (Table 2). Since the docking score of S-equol did not meet the threshold for refinement docking (−9 kcal/mol), this value was derived from the initial docking analysis. Notably, S-equol exhibited high binding stability towards TR*β*, second only to T3, suggesting that S-equol may potentially interact with the T3-binding site. Additionally, S-equol displayed comparable energy profiles for TR*α* and ER*α*, indicating structural characteristics that enable binding to TH and ERs. Collectively, these findings suggest that S-equol exhibits comparable binding affinity for TR*β* while also retaining affinity for ERs, providing a potential molecular basis for its modulatory effects on transcriptional activity across both receptor systems.

## 3. Discussion

In this study, we demonstrated that the plant-derived isoflavone metabolite, S-equol, enhances TR*β*-associated T3-induced transcriptional activity and neuronal morphogenesis. Although S-equol alone induced only a modest increase in transcriptional activity, co-exposure with T3 resulted in marked enhancement of transcriptional activity, neurite outgrowth, and wound closure. Furthermore, docking analyses revealed that S-equol exhibits moderate to high binding affinity for TR*β*, ER*α*, and ER*β*, with particularly strong binding stability toward TR*β*. Collectively, these findings suggest that S-equol may modulate TH action, potentially involving interaction between TR and ER signaling pathways. Although direct genetic or pharmacological disruption of TR*β* was not performed in this study, the minimal transcriptional activity induced by S-equol alone and requirement for T3 suggest that S-equol is unlikely to function as a direct TR*β* agonist, but rather modulates TR*β*-associated signaling in a cooperative manner. This interpretation is consistent with a role for S-equol as a signaling modulator rather than a primary receptor activator.

THs play essential roles in neural development, axonal elongation, and myelination [1,2], and non-genomic actions mediated by the MAPK and PI3K/Akt pathways have also been reported [11]. E2 similarly contributes to neuronal differentiation and the maintenance of neural plasticity, thereby regulating the expression of neurotrophic factors and synapse-related genes via ER*α* and ER*β* [3,4]. Because TRs and ERs belong to the nuclear receptor superfamily and share DNA-binding domains and coactivator complexes [5], functional interference and crosstalk between these receptors can occur.

Zhao et al. reported that T3 enhances ERE-dependent transcription in neuroblastoma cells [7], and Fujimoto et al. demonstrated that T3 increases ER expression and ERE activity in GH3 cells [8]. The enhancement of T3-induced transcriptional activity by S-equol observed in the present study is novel in that it suggests a potential interaction between TR and ER signaling pathways in the opposite direction; namely, the promotion of TR-associated transcription by an ER ligand-like compound.

However, the present study does not directly dissect the relative contributions of nuclear ERs and membrane-associated E2 signaling pathways. Therefore, although the involvement of ER-mediated mechanisms is strongly suggested by the known receptor selectivity and binding affinity of S-equol, further studies using receptor-specific antagonists or genetic approaches are needed to clarify the precise signaling pathways involved.

S-equol is produced through intestinal metabolism of daidzein [14,15], exhibits high affinity for ER*β*, and promotes neurite formation and exert neuroprotective effects [16,17]. Non-genomic signaling via GPR30/GPER1 has also been described [12]. Additionally, we reported that genistein and daidzein activate TR-dependent transcription [18], and that S-equol induces neurite formation through nuclear and membrane receptor pathways [16,17,19]. The present findings are consistent with these reports and further extend them by demonstrating a cooperative interaction between TR and ER signaling pathways.

In the docking analyses performed in this study, S-equol exhibited a relatively high binding energy towards TR*β* (≤7.0 kcal/mol), suggesting that it can form partially stable interactions within the T3-binding pocket. Because S-equol also displayed moderate affinity for ER*α* and ER*β*, it may function as a dual-acting modulator that may influence TR- and ER-related signaling pathways.

Morphological analyses further demonstrated that S-equol promotes neurite out-growth and wound closure under T3-treated conditions. Neurite formation is associated with increased expression of TR-related genes, such as MAP2 and βIII-tubulin [1,2], and is consistent with the induction of neural plasticity-related genes, including Bdnf and Syn1, via the E2 pathway [3,17]. Thus, S-equol may enhance neuronal morphogenesis under T3-treated conditions, potentially involving interaction between TR and ER signaling pathways. Under these conditions, S-equol alone did not further alter synapse-related gene expression despite its clear effects on neurite outgrowth, which may indicate a permissive or modulatory influence on neuronal differentiation rather than a direct transcriptional effect on synapse-related genes. This discrepancy suggests that the observed morphological effects may not be solely mediated by transcriptional regulation. It is possible that post-transcriptional mechanisms or cytoskeletal signaling pathways, such as those involving Rho GTPases or MAPK signaling, contribute to these effects. Further studies will be required to clarify these mechanisms. It should be noted that the wound healing assay may reflect both cell migration and proliferation. Although serum-free conditions were used to minimize proliferation, its contribution cannot be completely excluded. Therefore, the observed wound closure should be interpreted as reflecting cell motility-related processes rather than migration alone.

In line with this notion, qualitative immunocytochemical analyses performed in the context of the scratch assay showed increased detectability of Rho GTPase family members, including Cdc42, Rac1/2/3, and RhoA, in cells treated with S-equol and/or T3, whereas little signal was observed in control cells (Supplementary Figures S1–S3). Although these observations are qualitative, they are consistent with the involvement of cytoskeletal regulatory pathways in S-equol-associated morphological changes.

It should be noted that the present findings were obtained using Neuro-2a cells, a transformed neuroblastoma-derived model and C6 glioblastoma-derived cells, which are suitable for mechanistic analyses and retain functional TR and ER signaling pathways, but may not fully reflect all aspects of physiological neuronal differentiation in vivo. In this context, the effects of S-equol should be considered not only from a beneficial perspective but also in relation to potential developmental neurotoxicity. During the fetal and neonatal periods, sensitivity to thyroid hormones is particularly high [1,13], and even subtle alterations in TR activity may exert long-lasting effects on neural circuit formation. Although S-equol is an endogenous metabolite produced by the intestinal microbiota, its production capacity varies widely among individuals [15], and excessive exposure during development may modify neurodevelopmental processes. Therefore, S-equol can be regarded as a “nutritional endocrine modulator” with both beneficial effects and potential risks, warranting further investigation at the in vivo level.

Several limitations of this study should be acknowledged. The involvement of estrogen receptor (ER) signaling was not directly examined, as no experiments using ER antagonists or knockdown approaches were performed. In addition, the molecular docking analysis provides only a theoretical prediction of ligand–receptor interactions and does not account for protein dynamics or ligand availability in vivo. Furthermore, this study was conducted using transformed cell lines, which may not fully recapitulate the properties of primary neurons, and the concentrations of S-equol used may exceed typical physiological levels. Therefore, the findings should be interpreted with caution, and further studies using more physiologically relevant models are warranted.

## 4. Materials and Methods

### 4.1. Chemicals

S-equol was purchased from Cayman Chemical (Ann Arbor, MI, USA). 3,3′,5-Triiodo-L-thyronine (T3) was obtained from Sigma-Aldrich (St. Louis, MO, USA). All reagents used in this study were of analytical grade with a purity of ≥98%.

### 4.2. Cell Culture and Differentiation of Neuro-2a Cells

Mouse neuroblastoma-derived Neuro-2a cells and C6 rat glioma clonal cells were cultured in Dulbecco’s Modified Eagle’s Medium (DMEM) supplemented with 10% fetal bovine serum (FBS) and antibiotics (100 U/mL penicillin and 100 µg/mL streptomycin) at 37 °C in a humidified atmosphere containing 5% CO_2_. To remove endogenous hormones, FBS was treated prior to use by continuous mixing with 5% (*w*/*v*) AGXI-8 resin (Bio-Rad, Hercules, CA, USA) and powdered charcoal, as previously described [18]. Cells were cultured in medium supplemented with this treated FBS. Neuro-2a cells were seeded at a density of 1 × 10^5^ cells per well in 1 mL of DMEM containing 10% FBS on poly-L-lysine-coated 6-well or 24-well plates one day prior to experiments. Neuronal differentiation was induced by serum withdrawal as previously reported [20,21]. Briefly, the culture medium was replaced from DMEM containing 10% FBS to DMEM containing 1% FBS to initiate differentiation. On the following day, the medium was replaced with prewarmed DMEM containing 1% FBS with or without the indicated concentrations of S-equol and/or T3, and cells were cultured for 1–3 days. After treatment, cells were harvested for subsequent RT-qPCR or immunocytochemical analyses.

### 4.3. Luciferase Reporter Assay

TR*β*-dependent transcriptional activity was evaluated using a thyroid hormone response element (TRE)-driven luciferase reporter assay. Neuro-2a cells were seeded on poly-L-lysine-coated plates and transiently transfected with a human TR*β* expression plasmid together with a TRE–luciferase reporter plasmid using a commercially available transfection reagent, according to the manufacturer’s instructions. After transfection, cells were incubated in DMEM containing 1% FBS and treated with T3, S-equol, or their combination at the indicated concentrations for 24 h or for the indicated time periods. Following treatment, cells were lysed, and luciferase activity was measured using a luciferase assay system according to the manufacturer’s protocol. Luminescence was detected using a microplate luminometer.

Luciferase activity was normalized to the control group and expressed as relative transcriptional activity. Each experiment was performed in triplicate and repeated independently at least three times.

### 4.4. Immunocytochemistry and Quantification of Neurite Outgrowth

For immunocytochemical analysis, Neuro-2a cells were washed three times with phosphate-buffered saline (PBS), fixed with 4% paraformaldehyde (PFA), and blocked with 2% FBS in PBS. Cells were then simultaneously incubated with a mouse monoclonal anti-βIII-tubulin antibody (neuronal marker, 1:200; Sigma-Aldrich, St. Louis, MO, USA) and a primary antibody against synapsin I (presynaptic marker, 1:200; Sigma-Aldrich). F-actin was visualized using CytoPainter Phalloidin-iFluor 488 (Abcam, Cambridge, MA, USA), according to the manufacturer’s instructions. All staining reactions were performed under identical incubation conditions. After primary antibody incubation, cells were incubated with appropriate Alexa Fluor^®^-conjugated secondary antibodies. βIII-tubulin was detected using Alexa Fluor^®^ 405-conjugated secondary antibodies (blue), synapsin I was detected using Alexa Fluor^®^ 594-conjugated secondary antibodies (red), and F-actin was visualized by CytoPainter Phalloidin-iFluor 488 (green). Cell nuclei were counterstained with DAPI. Thus, βIII-tubulin, F-actin, and synapsin I were detected simultaneously using a three-channel fluorescence imaging approach. Fluorescence images were acquired using a laser scanning confocal microscope (LSM 880; Carl Zeiss Microscopy GmbH, Jena, Germany). Neurite length was quantified using ImageJ/Fiji 1.54f software (National Institutes of Health, Bethesda, MD, USA). Neurites were defined as βIII-tubulin-positive processes extending from the cell body, and neurite length was measured according to standard morphometric criteria.

### 4.5. In Vitro Wound Healing (Scratch) Assay

The in vitro wound healing (scratch) assay was performed as previously described [22]. C6 cells were seeded in 24-well plates and cultured until reaching confluence. Prior to scratch generation, cells were serum-starved in FBS-free DMEM for 6 h. A linear scratch was created in the cell monolayer using a 200 µL pipette tip, and detached cells were removed by washing with PBS (−). Serum-free DMEM containing S-equol and/or T3 was added to each well, followed by incubation for an additional 24 h. Live-cell staining was performed at 0 h and 24 h using Cellstain-Hoechst 33258 solution (Dojindo Laboratories, Kumamoto, Japan) according to the manufacturer’s protocol. Images of the scratched areas were acquired at identical positions at 0 h and 24 h using a fluorescence microscope (BZ-9000; Keyence, Osaka, Japan). Wound closure was evaluated at the wound edge, and the wound closure rate was calculated as the ratio of the wound closure distance to the initial wound width.

### 4.6. Quantitative Real-Time PCR (RT-qPCR)

Total RNA was extracted from Neuro-2a cells using a commercially available RNA extraction kit according to the manufacturer’s instructions. The concentration and purity of isolated RNA were determined spectrophotometrically. Complementary DNA (cDNA) was synthesized from equal amounts of total RNA using a ReverTra Ace (TOYOBO, Osaka, Japan). Quantitative real-time PCR was performed using gene-specific primers for *Dlg4*, *Syn1*, *Syp*, *Camk2b*, and *Bdnf* (primer sequences are listed in Table S1) with a real-time PCR detection system and a THUNDERBIRD SYBR qPCR Mix (TOYOBO). The amplification conditions were set according to the manufacturer’s recommendations. Relative gene expression levels were calculated using the ΔΔCt method, with *Gapdh* used as an internal control. Gene expression values were normalized to *Gapdh* and expressed as fold changes relative to the control group. Each sample was analyzed in triplicate, and experiments were independently repeated at least three times.

### 4.7. In Silico Ligand–Receptor Binding Analysis

In silico molecular docking analysis was performed as described previously [18,23], with slight modifications. All in silico calculations were performed using a workstation with AMD Ryzen 7 3800x 8-Core @ 3.9 GHz, 32 GB DDR4 3200 MHz, AMD Radeon RX 5700 XT 8 GB, running on a Windows 11 professional operating system. Molecular structures for T3 (PubChem CID 5920), S-equol (PubChem CID 91469), and E2 (PubChem CID 5757) were downloaded from PubChem (https://pubchem.ncbi.nlm.nih.gov/, last accessed 26 December 2025) in sdf format. The crystal structures of the TR-LBD (PDB IDs: 4LNX, 4LNW, 3JZB, 3GWS, 2PIN, and 1NAX) and ER-LBD (PDB IDs: 1A52, 1X7R, 3ERT, 1QKM, 3OLL, and 5TOA) were downloaded from the Protein Data Bank (PDB) (http://www.rcsb.org/pdb/home/home.do, last accessed 26 December 2025) in PDB format. The three-dimensional structure of TR-LBD, ER-LBD, and ligands files were opened and modified with BIOVIA Discovery Studio structure-based design software, version 2017 R2 (BIOVIA/Accelrys Inc., San Diego, CA, USA (https://discover.3ds.com/discovery-studio-visualizer-download, last accessed 26 December 2025)). Water molecules and other substructures (bound molecules/ligand molecules) were removed from the coordinate file before docking. The unliganded TR-LBD and ER-LBD were used for the individual dockings of T3, E2, and S-equol. Polar hydrogen atoms were added to the 3D structure of the TR-LBD or ER-LBD and the input file was generated in the PDBQT format, which contained the structure of the TR-LBD or ER-LBD, using AutoDockTools of MGLTools version 1.5.6 (https://ccsb.scripps.edu/projects/docking/, last accessed 26 December 2025). The coordinates for docking were determined through a grid box using the PyRx-Python Prescription 0.8 Virtual Screening software for Computer-Aided Drug Design (https://pyrx.sourceforge.io/, last accessed 26 December 2025), using AutoDock 4 and AutoDock Vina as docking software [24]. A blind docking strategy was utilized in order to include the entire possible binding site for ligands. For more reliable results, refinement docking experiments with repetitions of 30 runs were performed with complexes which had high affinity scores (lower than −9 kcal/mol). Since the docking score of S-equol did not meet this criterion, the value reported for S-equol corresponds to the initial docking result. LigPlot+ v.1.4 (http://www.ebi.ac.uk/thornton-srv/software/LigPlus/, last accessed 26 December 2025) and BIOVIA Discovery Studio were used to determine the interactions between the TR-LBDs and the ligands in complexes with the best affinity scores. The binding affinity was expressed as the binding free energy (kcal/mol).

### 4.8. Statistical Data Analysis

Data were analyzed using one or two way ANOVA. A post hoc comparison was performed using Bonferroni’s test. All data in the text and figures are expressed as the mean and SEM. Statistical significance was set at *p* < 0.05.

## 5. Conclusions

This study demonstrated that the plant-derived isoflavone metabolite S-equol enhances TR*β*-associated T3-induced transcriptional activity and promotes neurite outgrowth and wound closure. Additionally, co-exposure to S-equol in the presence of T3 enhanced the expression of synapse-related genes (*Dlg4*, *Syn1*, *Syp*, *Camk2b*, and *Bdnf*), suggesting that S-equol may also contribute to synaptic plasticity. Furthermore, S-equol exhibited moderate to high binding affinity for TR*β*, ER*α*, and ER*β*, suggesting that it may modulate TH action, potentially involving interaction between TR and ER signaling pathways. Although S-equol exerts beneficial neuroprotective effects, it also functions as an endocrine-modulating compound capable of altering the fine regulation of TH action during development, highlighting the need for evaluation from both physiological and toxicological perspectives.

## Figures and Tables

**Table 1:** The binding affinities of TR-LBD for T3 and ER-LBD for Estradiol. Results of the Refinement Docking Experiments with AutoDock Vina version 1.2.5. The binding affinities of TR*α* (4LNX; 4LNW; 3JZB) and TR*β* (3GWS; 2PIN; 1NAX) for T3, as well as ER*α* (1A52; 1X7R; 3ERT) and ER*β* (1QKM; 3OLL; 5TOA) for E2, were ≤7.0 kcal/mol.

T3		Estradiol	
PDB-ID	Affinity	PDB-ID	Affinity
4LNX (TR*α*-LBD)	−9.4	1A52 (ER*α*-LBD)	−8.5
4LNW (TR*α*-LBD)	−9.1	1X7R (ER*α*-LBD)	−9.8
3JZB (TR*α*-LBD)	−9.2	3ERT (ER*α*-LBD)	−9.0
3GWS (TR*β*-LBD)	−9.8	1QKM (ER*β*-LBD)	−10.1
2PIN (TR*β*-LBD)	−9.5	3OLL (ER*β*-LBD)	−9.3
1NAX (TR*β*-LBD)	−10.0	5TOA (ER*β*-LBD)	−11.1

**Table 2:** The binding affinities of S-equol with TR-LBD or ER-LBD. S-equol, which exhibits a similar binding pose under blind docking procedures, also shows binding affinities ≤7.0 kcal/mol for both TR and ER.

TR*α*-LBD		TR*β*-LBD		ER*α*-LBD		ER*β*-LBD	
PDB-ID	Affinity	PDB-ID	Affinity	PDB-ID	Affinity	PDB-ID	Affinity
4LNX	−9.6	3GWS	−9.6	1A52	−9.1	1QKM	−8.8
4LNW	−9.7	2PIN	−9.9	1X7R	−9.2	3OLL	−7.1
3JZB	−9.3	1NAX	−7.8	3ERT	−8.9	5TOA	−9.0

## Data Availability

The data presented in this study are available on request from the corresponding author.

## Supplementary Materials

The following supporting information can be downloaded at: https://www.mdpi.com/article/10.3390/ijms27073253/s1.

## Abbreviations

The following abbreviations are used in this manuscript:

TRs	Thyroid hormone receptors
THs	Thyroid hormone
TREs	Thyroid hormone response elements
E2	Estrogen
ER	E2 receptors
MAPK	Mitogen-activated protein kinase
ERE	E2 response element
GPER1	G protein-coupled E2 receptor 1
LBD	Ligand-binding domain
