# Supplementary material for: S-equol Modulates T3-Induced Transcription and Neurite Outgrowth in Neuronal Cells
Source: Int J Mol Sci. 2026 Apr 3;27(7):3253. doi: 10.3390/ijms27073253 (PMC13073576; doi:10.3390/ijms27073253)
Supplement: Supplementary file 1 [file ijms-27-03253-s001.zip › Supplementary Figure S2.pdf]

Figure S2

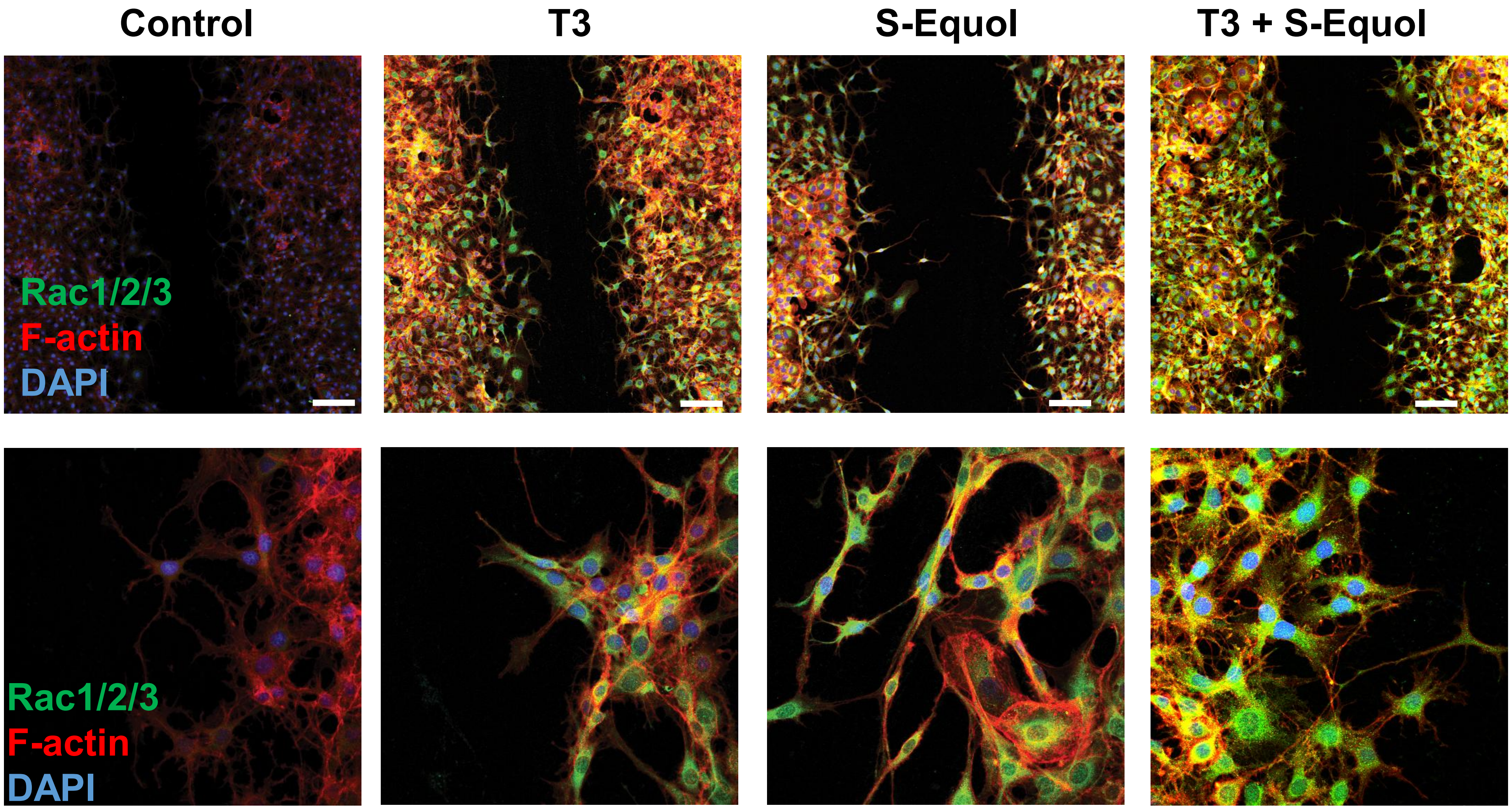

Supplementary Figure S2. Immunocytochemical detection of Rac1/2/3 in Neuro-2a cells following treatment with S-equol and/or T3.

Neuro-2a cells treated with vehicle (control), S-equol, T3, or their combination were subjected to immunocytochemical staining for Rac1/2/3 (green) and F-actin (red), with nuclei counterstained with DAPI (blue). Representative fluorescence images are shown in the upper panels, with corresponding magnified views in the lower panels. Rac1/2/3 immunoreactivity was scarcely detectable in control cells but was increased in cells treated with S-equol and/or T3. Scale bar=100 μm.
