# Supplementary material for: S-equol Modulates T3-Induced Transcription and Neurite Outgrowth in Neuronal Cells
Source: Int J Mol Sci. 2026 Apr 3;27(7):3253. doi: 10.3390/ijms27073253 (PMC13073576; doi:10.3390/ijms27073253)
Supplement: Supplementary file 1 [file ijms-27-03253-s001.zip › Supplementary Figure S3.pdf]

Figure S3

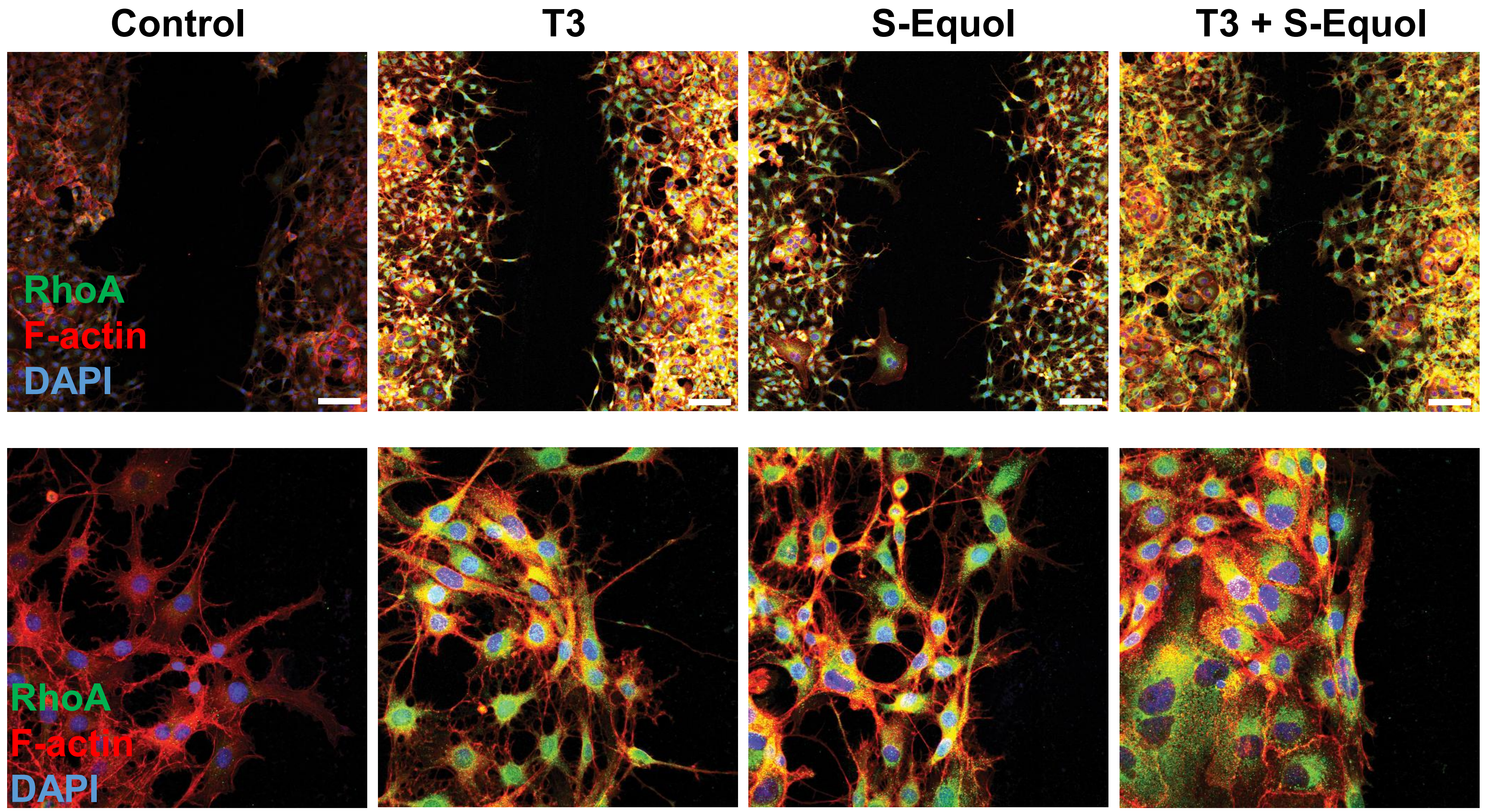

Supplementary Figure S3. Immunocytochemical detection of RhoA in Neuro-2a cells following treatment with S-equol and/or T3.

Neuro-2a cells treated with vehicle (control), S-equol, T3, or their combination were subjected to immunocytochemical staining for RhoA (green) and F-actin (red), with nuclei counterstained with DAPI (blue). Representative fluorescence images are shown in the upper panels, with corresponding magnified views in the lower panels. RhoA immunoreactivity was scarcely detectable in control cells but was increased in cells treated with S-equol and/or T3. Scale bar=100 μm.
