# Supplementary material for: S-equol Modulates T3-Induced Transcription and Neurite Outgrowth in Neuronal Cells
Source: Int J Mol Sci. 2026 Apr 3;27(7):3253. doi: 10.3390/ijms27073253 (PMC13073576; doi:10.3390/ijms27073253)
Supplement: Supplementary file 1 [file ijms-27-03253-s001.zip › Table S1.pdf]

Supplementary Table

Table S1. Primer sequences.

Gene	Sense	Antisense
Dlg4 (Mouse)	GGTCAACGACAGCATCCTG	ATGACGTAGAGGCGAACGATG
Syn1 (Mouse)	TGTGCGTGTCCAGAAGATTG	ACATGGCAATCTGCTCAAGC
Syp (Mouse)	TTTGCCATCTTCGCCTTTGC	GGGTGCATCAAAGTACACTTGG
Camk2b (Mouse)	TGCAAGGAGGAAGCTCAAGG	CTGTTTGTCTGGGGCTTGAC
Bdnf (Mouse)	ATCCAAAGGCCAACTGAAGC	ATTGGGTAGTTCGGCATTGC
Gapdh (Mouse)	TGCGACTTCAACAGCAACTC	ATGTAGGCCATGAGGTCCAC
